# Time trends in demand for family planning satisfied: analysis of 73 countries using national health surveys over a 24-year period

**DOI:** 10.7189/jogh.09.020423

**Published:** 2019-12

**Authors:** Franciele Hellwig, Carolina VN Coll, Fernanda Ewerling, Aluisio JD Barros

**Affiliations:** 1 International Center for Equity in Health, Federal University of Pelotas, Pelotas, RS, Brazil; 2 Postgraduate Program in Epidemiology, Federal University of Pelotas, Pelotas, RS, Brazil

## Abstract

**Background:**

Universal access to family planning is key to extend its health and economic benefits worldwide. Our aim was to track progress in demand for family planning satisfied with modern methods (mDFPS) and its inequalities in low- and middle-income countries (LMICs).

**Methods:**

Analyses were based on Demographic and Health Surveys and Multiple Indicator Cluster Surveys carried out between 1993 and 2017 in 73 LMICs, using data for married women aged 15-49 years. We estimated trends in mDFPS coverage by country and world region and evaluated trends in wealth-based inequalities. The analyses pooling all countries together were stratified by wealth quintiles, area of residence and woman’s age. mDFPS coverage in 2030 for each country was predicted using a linear model.

**Results:**

Overall, mDFPS increased and poor-rich gaps narrowed. Eastern & Southern Africa showed an average increase of 1.5 percentage points (p.p.) a year, being the region with the fastest progress. West & Central Africa had an increase in mDFPS of 1 p.p. a year but current coverage is still below 40%. Generally, inequalities were reduced, except for West & Central Africa and Europe & Central Asia where almost no change was observed. The country with the fastest progress in mDFPS was Rwanda, with an increase of 5 p.p./y, while Timor Leste had the fastest reduction in absolute inequality, less 3.8 p.p./y. Inequalities by area of residence were reduced, but large gaps remain. A similar trend was observed for different age groups. If the current trend is not accelerated, 44 countries will not achieve universal coverage in mDFPS by 2030.

**Conclusions:**

Generally, mDFPS is increasing and inequalities are decreasing. However, progress is slow in some regions, especially West & Central Africa, where low coverage is combined with high levels of inequalities. Efforts to increase family planning coverage must be prioritized in countries where progress is slow or inexistent.

Family planning has changed the lives of women, families and countries. Modern contraception allows women to fulfil their reproductive rights. It decreases child mortality and gives families the opportunity to reduce their size and invest more in each child [1,2]. It also gave whole countries the opportunity to benefit from what was called the demographic dividend – suddenly a country had a large working force with a relative smaller number of younger and older people to cater to [3-5]. However, in some regions and countries, we still have low usage of contraceptives for family planning with sustained high fertility rates [6]. The main barriers to increase the uptake of family planning methods are related to social norms, family or partner disapproval and concerns about side effects [7-9]. Social norms – such as expectations of a newly married woman to prove the couple’s fertility, marriage at early ages, social gains with having children – are a key aspect in many of the countries where use of contraceptives is low [10-13]. The availability of family planning support and supplies also plays an important role in this scenario [8,10,11].

The Sustainable Development Goals (SDGs) 3 (ensure good health and well-being) and 5 (achieve gender equality and empower all women and girls) encompass universal sexual and reproductive health. Family planning can also help to achieve other SDGs [3,14,15] by enabling women to stay employed or to complete education, reducing poverty and gender inequalities [3,16,17]. Through increases on human capital and social interaction, education may lead women to change their expectations about the future and to improve their autonomy and access to sexual and reproductive health services, playing an important role to reduce social and cultural barriers and increase modern contraceptive use [18-20]. Employed women tend to have more decision power about household spending and make a larger share of expenditure to go to essential basic goods as food, resulting in improved family nutrition [3,17]. Women who use contraception to plan their families generally have less children and larger spacing between pregnancies [4], preventing high-risk pregnancies and unsafe abortions, which improves maternal and child health and survival [4,5,17,21,22]. Therefore, investing in family planning programs is essential for progress towards the SDGs.

Since the 1960s, many efforts have been made to reach disadvantaged populations and increase the use of family planning methods [23]. As a result, contraceptive use increased from 10% to 60% globally [24], but, the use of contraception is still low in some regions. This is better understood with the indicator for demand for family planning satisfied (DFPS), that is the proportion of women in need of contraception actively using them. A recent analysis of low- and middle-income countries (LMICS) showed that in West & Central Africa a mere 33% of women had their need for family planning satisfied with modern contraceptives [25]. In Easter Europe the authors reported DFPS coverage below 50% but in a completely different scenario – where fertility is low, and the low coverage is explained by the high proportion of traditional contraceptive methods use [25]. This study also showed that some groups are harder to be reached by family planning strategies. Use of contraceptives is lower among women in the poorest wealth quintiles, living in rural areas, who are younger and less educated [22,25]. These disparities are most striking in West & Central Africa and East & Southern Africa, where the greatest disparities are in terms of wealth [26].

Even though demand for family planning satisfied increased worldwide, the progress has been uneven and slow in some regions [4,27,28]. A recent report showed that DFPS increased significantly during the 1990s in all regions, however, this increase has been slower since 2000. In the 2010s, Asian regions achieved a contraceptive use prevalence higher than 60%, close to Latin America and Eastern Europe levels. Besides those regions, a significant increase was observed only in Eastern and Southern Africa [29,30].

Monitoring trends in national DFPS as well as trends according to key characteristics to strengthen family planning programs is essential to achieve universal coverage worldwide. In this study we explored trends in family planning in LMICs, using demand for family planning satisfied with modern contraceptive methods (mDFPS) as our main indicator. To identify regions and countries where increase is slower than the needed, we looked at individual countries and at world regions, using the countries where data are available from national health surveys. To guarantee the comparability among countries, we use data collected and analyzed in a standardized way. We also assessed the trends in inequalities of mDFPS according to wealth, woman’s age, and area of residence to identify population subgroups who are lagging behind over time.

## METHODS

To evaluate trends in mDFPS we used data from Demographic and Health Survey (DHS) and Multiple Indicator Cluster Survey (MICS) programs. DHS and MICS use highly standardized questionnaires, methodology and sampling design [31,32]. All of these surveys are representative at national level, based on multistage cluster samples and are sanctioned by either DHS or MICS programs and the countries’ governments. The surveys included in the analysis are presented in Table S1 in **Online Supplementary Document** and comprise 281 household surveys from 73 LMICs. In order to obtain recent and reliable estimates, we selected countries that had at least two surveys carried out five or more years apart, with the most recent survey since 2010. The surveys included in the analysis cover the period from 1993 to 2017. mDFPS was estimated in a standardized way to guarantee comparability across time points and countries.

More recently, studies on family planning have been using all sexually active women in the sample instead of partnered women only, since sexually active women, partner or not, may be in need of contraception. However, this would lead to the exclusion of all countries from Middle East & North Africa, and several countries from other world regions, totaling 62 surveys from 24 countries. Therefore, to maximize the number of surveys in the analysis, a key issue in trend analyses, we studied women that were married or in a union (hereafter referred to as *married women*, for simplicity), aged 15-49 years. Trends in countries with information for all sexually active women are presented in the supplementary material, as well as a comparison between mDFPS among married women and all sexually active women for surveys where such information is available.

### Outcome

mDFPS was defined here as the proportion of married women of reproductive age (15-49 years old) in need of contraception that are currently using a modern contraceptive method. The denominator of mDFPS, women in need of contraception, was defined as women who are fecund and do not want to become pregnant within the next two years or are unsure if or when they want to become pregnant. Pregnant women at the time of the survey with a mistimed or unwanted pregnancy were also considered in need of contraception. Women were considered infecund and were excluded from the denominator if they were menopausal; had had a hysterectomy; had never menstruated; had had the last period more than 6 months ago and were not postpartum amenorrhoeic; said they cannot get pregnant; or if they were married for five or more years, had never used contraception and had no children in the previous five years [33,34].

In the numerator we had women who were using modern contraceptives, or whose partners were. There is not a consensus on which contraceptive methods are classified as modern in the literature. We used the definition of *technological products or medical procedures that affect natural reproduction* [35]. According to this definition, modern methods include contraceptive pills, condoms (male and female), diaphragms, spermicidal agents (foam/jelly), injectables, emergency contraception, intrauterine devices (IUD), hormone implants and sterilization (male and female). Withdrawal, any method based on calendar and lactational amenorrhea were not considered modern methods.

### Analysis

Trends in mDFPS were estimated for each country nationally and globally according to woman’s age (15-17 years; 18-19 years; 20-49 years), area of residence (urban or rural) and wealth quintiles of the wealth index provided with the surveys, which was calculated through principal component analyses of household assets and building characteristics (Q1 being the poorest and Q5 the wealthiest quintile).

We also calculated two wealth-based inequality measures, the slope index of inequality (SII) for absolute inequality and the concentration index (CIX) for relative inequality. The SII represents the difference between the coverage for the top and the bottom of the wealth scale, expressed in percentage points. The coverage for the extremes of the wealth distribution was calculated using logistic regression [36]. The CIX estimates by how much an attribute is concentrated towards the poorer or the richer. In a Lorenz curve, where we plot the cumulative frequency of the attribute against the rank of each individual in the wealth distribution, the CIX is represented by twice the area between the curve and diagonal. The curve defined in a completely equitable situation will lie on the diagonal and the area (and the CIX) will be zero. Both measures are expressed on a scale from –100 to +100, in which 0 represents perfect equality. A positive measure indicate that the coverage is higher among the wealthier, also called a pro-rich distribution, while a negative value means the coverage is concentrated towards the poor [37]. Absolute and relative measures are complementary, while the absolute inequality shows the gap existing between subgroups, the relative inequality gives an idea of how unfair these inequalities are [36]. Using both types of measures is even more important when analyzing trends in inequalities, as one of them can increase while the other decreases over time, depending on the rate of progress achieved by the different subgroups in the population [37].

National average annual absolute change (AAAC) for the SII, CIX and mDFPS was estimated using variance weighted least squares regression and global and regional AAAC was estimated using meta-regression. Regional trends are presented according to the UNICEF classification: West & Central Africa, Eastern & Southern Africa, Middle East & North Africa, Europe & Central Asia, South Asia, East Asia & Pacific, and Latin America & Caribbean. We estimated AAAC in mDFPS and in inequality measures for mDFPS at global level with and without taking into account country size. We decided to present our results based on unweighted means, given that we do not have information on all countries from each region, so these results could not be interpreted as representative of the region. A comparison of both unweighted and weighted means is presented in the Table S2 in **Online Supplementary Document**. Global trends in mDFPS by woman’s age, area of residence, wealth quintiles, and wealth-based inequality measures are unweighted means.

Considering the SDG targets to achieve universal family planning coverage by 2030, we also predicted each country’s level of mDFPS in 2030 using the same linear model fitted to estimate AAAC. Following the recommended benchmark, we considered universal access at least 75% of mDFPS coverage [5,38].

As MICS and DHS have complex sample strategies, all estimates considered the sample design, including clusters, strata and sample weights. All analyses were conducted using Stata (StataCorp, College Station, TX, USA). All analyses relied on publicly available, anonymized databases and ethical clearance was obtained by the national agencies responsible for the conduction of each survey.

## RESULTS

We analyzed 31% of the LMIC in the Middle East & North Africa region (which represents 42% of the total population of the region), 88% of the West & Central Africa countries (99% of the population), 71% of Eastern & Southern Africa (88% of the population), 57% of Europe & Central Asia (26% of the total population), 71% of South Asia (98% of the total population), 26% of East Asia & Pacific (24% of the population), and 40% of the Latin America & Caribbean countries (25% of the total population of the low- and middle-income countries in the region).

**Figure 1:**
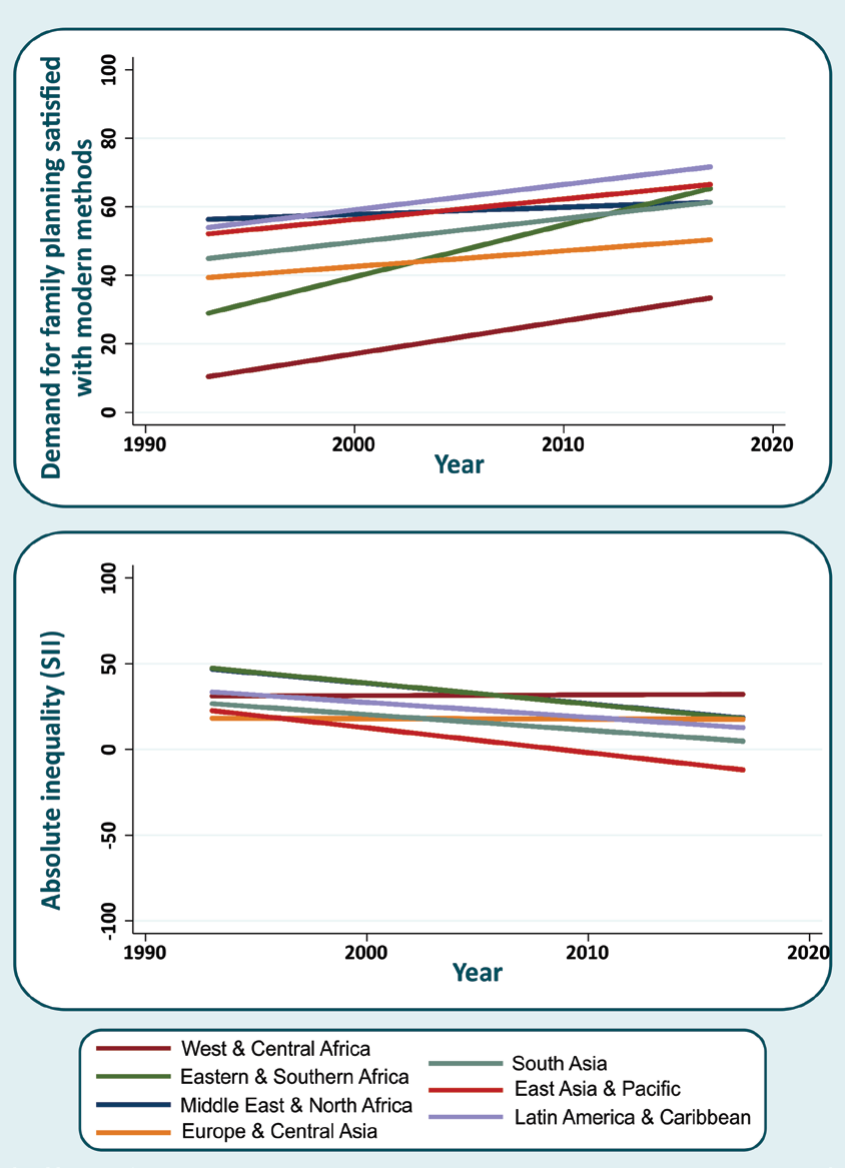
Trends in demand for family planning satisfied with modern methods (mDFPS) and in absolute inequality (SII) in mDFPS according to world region.

Between 1993 and 2017, mDFPS increased in all world regions, but at different rates (**Figure 1**). The fastest progress was observed in Eastern & Southern Africa countries (1.5 percentage points (p.p.) per year, on average). In the 1990s, the countries in the region had the second lowest mDFPS coverage, and currently their coverage is reaching the top positions. The regions with the highest coverage in the 1990s were Latin America & Caribbean and Middle East & North Africa. However, information was available for only five and two countries from each region, respectively, during the 1990s. The slowest progress was observed in Europe & Central Asia (0.5 p.p. per year, on average). Other regions with slow progress were Latin America & Caribbean, East Asia & Pacific, and South Asia, however, these regions already presented the highest levels of coverage at the start of the study period and low inequality. On the low coverage side, despite the improvement observed (increase of 1 p.p. per year, on average), the countries in West & Central Africa still presented mDFPS below 40%. AAAC in mDFPS by country is presented in **Table 1**. Rwanda (Eastern & Southern Africa) was the country with the fastest progress (4.6 p.p. per year, on average). Other countries with very good progress were Sierra Leone in West & Central Africa, and Ethiopia in Eastern & Southern Africa, with AAAC of 3.6 and 3.0 p.p. per year, on average, respectively. The current level of mDFPS should, however, be considered along with the change. Many countries had an improvement slower than 1 p.p. a year, but several already had a reasonable level of mDFPS. Among 13 countries already presented mDFPS above 75% (Colombia, Cuba, Dominican Republic, Eswatini, Egypt, Honduras, Indonesia, Kazakhstan, Lesotho, Namibia, South Africa, Turkmenistan, and Zimbabwe) while projections for 2030 reveal that another 17 countries will be able to reach this threshold by 2030. The remaining 43 countries had low mDFPS and increase in coverage was slow or null, suggesting that they will not achieve 75% coverage by 2030 if the current trend is not accelerated (**Table 1**).

**Table 1:** Average annual absolute change (AAAC) in demand for family planning satisfied with modern methods (mDFPS) and mDFPS projection for 2030 by country

Country	Year	AAAC			Projected mDFPS in 2030
**mDFPS (*P* value)**	SII* (*P* value)	CIX* (*P* value)
**All countries**		**0.91 (<0.001)**	**-0.69 (<0.001)**	**-0.47 (<0.001)**	—
**Middle East & North Africa**		**0.21 (0.131)**	**-1.19 (<0.001)**	**-0.36 (<0.001)**	—
Egypt	1995-2014	0.43 (<0.001)	-1.19 (<0.001)	-0.27 (<0.001)	>75
Iraq	2006-2011	-1.05* (<0.001)	-	-	<50
Jordan	1997-2017	0.10 (0.081)	-0.97 (<0.001)	-0.38 (<0.001)	50-75
Yemen	2006-2013	-0.29 (0.458)	-2.62 (<0.001)	-1.02 (<0.001)	<50
**West & Central Africa**		**0.96 (<0.001)**	**-0.04 (<0.001)**	**-0.10 (<0.001)**	—
Benin	1996-2014	0.61 (<0.001)	0.14 (0.400)	-0.76 (<0.001)	<50
Burkina Faso	1998-2010	2.01 (<0.001)	0.76 (0.021)	-2.15 (<0.001)	>75
Central African Republic	1994-2010	0.90 (<0.001)	3.00 (<0.001)	0.51 (0.121)	<50
Cameroon	1998-2014	0.95 (<0.001)	0.70 (0.006)	-0.40 (0.024)	50-75
Chad	1996-2014	0.15 (0.016)	-0.94 (0.001)	-1.79 (<0.001)	<50
Congo Brazzaville	2005-2014	1.84 (<0.001)	1.09 (0.054)	0.-0.69 (0.063)	50-75
Congo DR	2007-2013	0.38 (0.117)	0.10 (0.900)	-1.15 (0.215)	<50
Cote d'Ivoire	1994-2016	0.89 (<0.001)	0.26 (0.895)	-1.01 (<0.001)	<50
Gabon	2000-2012	1.12 (<0.001)	-0.22 (0.720)	-0.65 (0.047)	50-75
Ghana	1993-2014	1.04 (<0.001)	-1.04 (<0.001)	-1.12 (<0.001)	50-75
Guinea	1999-2016	0.16 (0.046)	-0.23 (0.468)	-0.65 (0.014)	<50
Guinea Bissau	2006-2014	2.01 (<0.001)	-0.47 (0.526)	-2.76 (0.014)	50-75
Liberia	2007-2013	2.52 (<0.001)	-1.56 (0.218)	-2.00 (0.014)	>75
Mali	1995-2015	0.84 (<0.001)	0.76 (<0.001)	-0.74 (<0.001)	<50
Mauritania	2007-2015	0.62 (0.002)	1.07 (0.007)	-0.04 (0.914)	<50
Niger	1998-2012	0.64 (<0.001)	0.19 (0.498)	-1.06 (0.001)	<50
Nigeria	1999-2016	0.21 (0.005)	0.03 (0.852)	-0.18 (0.309)	<50
Sao Tome and Principe	2008-2014	1.38 (0.003)	-1.44 (0.264)	-0.36 (0.446)	50-75
Senegal	1997-2017	1.66 (<0.001)	-0.44 (0.029)	-1.72 (<0.001)	50-75
Sierra Leone	2008-2013	3.60 (<0.001)	-2.31 (0.047)	-4.41 (<0.001)	>75
Togo	1998-2013	1.22 (<0.001)	-0.46 (0.123)	-1.40 (<0.001)	50-75
**Eastern & Southern Africa**		**1.50 (<0.001)**	**-1.17 (<0.001)**	**-1.10 (<0.001)**	
Burundi	2010-2016	0.95 (<0.001)	-2.35 (0.003)	-1.29 (<0.001)	50-75
Comoros	1996-2012	0.33 (0.008)	-0.60 (0.148)	-0.62 (0.045)	<50
Eswatini	2006-2014	2.62 (<0.001)	-2.11 (0.001)	-0.65 (<0.001)	>75
Ethiopia	2000-2016	2.95 (<0.001)	0.05 (0.686)	-2.00 (<0.001)	>75
Kenya	1993-2014	1.41 (<0.001)	-1.11 (<0.001)	-0.75 (<0.001)	>75
Lesotho	2004-2014	2.45 (<0.001)	-3.43 (<0.001)	-1.44 (<0.001)	>75
Malawi	2000-2015	2.30 (<0.001)	-1.33 (<0.001)	-0.62 (<0.001)	>75
Mozambique	1997-2015	1.40 (<0.001)	-0.64 (0.058)	-1.58 (<0.001)	<50
Namibia	2000-2013	0.71 (<0.001)	-1.66 (<0.001)	-0.51 (<0.001)	>75
Rwanda	2000-2014	4.57 (<0.001)	-1.74 (<0.001)	-1.93 (<0.001)	>75
South Africa	1998-2016	0.02 (0.795)	-1.91 (<0.001)	-0.43 (<0.001)	>75
Tanzania	1996-2015	1.07 (<0.001)	-1.45 (<0.001)	-1.07 (<0.001)	50-75
Uganda	1995-2016	1.53 (<0.001)	-1.09 (<0.001)	-1.71 (<0.001)	50-75
Zambia	1996-2013	1.94 (<0.001)	-1.07 (<0.001)	-1.36 (<0.001)	>75
Zimbabwe	1994-2015	1.04 (<0.001)	-1.51 (<0.001)	-0.41 (<0.001)	>75
**Europe & Central Asia**		**0.46 (0.016)**	**-0.02 (0.900)**	**-0.05 (0.565)**	
Albania	2008-2017	-0.72 (<0.001)	0.28 (0.244)	-0.20 (0.307)	<50
Armenia	2000-2015	0.82 (<0.001)	0.24 (0.391)	-0.20 (0.249)	50-75
Bosnia and Herzegovina	2006-2011	0.09 (0.847)	-0.55 (0.741)	-0.59 (0.612)	<50
Kazakhstan	1995-2015	1.12 (<0.001)	-0.17 (0.438)	-0.07 (0.194)	>75
Kyrgyzstan	1997-2014	-0.31 (0.010)	-0.33 (0.346)	-0.08 (0.364)	50-75
Macedonia	2005-2011	0.82 (0.130)	2.67 (0.134)	0.82 (0.509)	<50
Moldova	2005-2012	1.42 (<0.001)	-0.81 (0.249)	-0.40 (0.045)	>75
Montenegro	2005-2013	1.41* (0.003)	1.43 (0.232)	0.16 (0.797)	50-75
Serbia	2005-2014	0.35 (0.118)	0.33 (0.634)	0.00 (0.990)	<50
Tajikistan	2005-2017	-0.43 (0.005)	-0.57 (0.245)	-0.18 (0.235)	<50
Turkmenistan	2006-2015	0.64 (<0.001)	-0.05 (0.808)	-0.03 (0.768)	>75
Ukraine	2005-2012	-0.01 (0.979)	-0.55 (0.562)	0.96 (<0.001)	50-75
**South Asia**		**0.68 (0.001)**	**-0.91 (<0.001)**	**-0.32 (<0.001)**	
Bangladesh	1993-2014	1.24 (<0.001)	-0.52 (<0.001)	-0.14 (<0.001)	>75
India	1998-2015	0.26 (<0.001)	-0.55 (<0.001)	-0.14 (<0.001)	>75
Maldives	2009-2016	-1.83 (<0.001)	0.74 (0.398)	0.52 (0.234)	<50
Nepal	1996-2016	0.82 (<0.001)	-2.06 (<0.001)	-0.80 (<0.001)	50-75
Pakistan	2006-2017	0.75 (<0.001)	-1.46 (<0.001)	-0.78 (<0.001)	50-75
**East Asia & Pacific**		**0.60 (<0.001)**	**-1.4 (<0.001)**	**-0.55 (<0.001)**	
Cambodia	2000-2014	1.59 (<0.001)	-2.82 (<0.001)	-1.13 (<0.001)	>75
Indonesia	1994-2012	-0.01 (0.713)	-0.63 (<0.001)	-0.13 (<0.001)	>75
Mongolia	2005-2013	-0.34 (0.010)	-0.80 (0.071)	-0.19 (0.047)	50-75
Philippines	1993-2017	0.74 (<0.001)	-1.55 (<0.001)	-0.67 (<0.001)	50-75
Timor Leste	2009-2016	0.99 (<0.001)	-3.82 (<0.001)	-1.70 (<0.001)	50-75
Vietnam	1997-2013	0.49 (<0.001)	-0.66 (0.009)	-0.14 (0.020)	>75
**Latin America & Caribbean**		**0.74 (<0.001)**	**-0.87 (<0.001)**	**-0.46 (<0.001)**	
Belize	2006-2015	1.92 (<0.001)	0.19 (0.857)	-0.15 (0.475)	>75
Colombia	1995-2015	0.82 (<0.001)	-1.00 (<0.001)	-0.26 (<0.001)	>75
Cuba	2006-2014	-0.02 (0.864)	-	-	>75
Dominican Republic	1996-2014	0.46 (<0.001)	-0.32 (0.029)	-0.08 (0.010)	>75
Guatemala	1995-2014	0.95 (<0.001)	-1.90 (<0.001)	-1.01 (<0.001)	>75
Guyana	2006-2014	0.22 (0.421)	0.92 (0.281)	0.19 (0.501)	50-75
Haiti	1994-2016	0.98 (<0.001)	- -0.93 (<0.001)	-0.66 (<0.001)	50-75
Honduras	2005-2011	1.28 (<0.001)	-2.26 (<0.001)	-0.55 (<0.001)	>75
Peru	1996-2016	0.50 (<0.001)	-0.78 (<0.001)	-0.31 (<0.001)	50-75
Trinidad and Tobago	2006-2011	1.61 (<0.001)	1.40 (0.305)	0.27 (0.464)	>75

SII – Slope Index of Inequality, CIX – Concentration Index of Inequality.

*Negative values indicate reduction in inequality, which means that the most recent survey shows a value that is closer to the equity situation than the earlier survey.

Absolute (SII) and relative (CIX) inequalities in mDFPS were substantially reduced worldwide in the period analyzed (**Table 1**). While all regions reduced the absolute inequalities, the reduction patterns varied greatly (**Table 1** and **Figure 1**). The countries in East Asia & Pacific presented the highest reduction in absolute inequalities (1.4 p.p. per year, on average), with negative indicator values in the most recent years, which means that the poorest women achieved higher mDFPS coverage than the wealthiest. Middle East & North Africa and Eastern & Southern Africa also showed a high reduction in the SII (1.2 p.p. per year, on average). West & Central Africa and Europe & Central Asia showed very small positive changes in both inequality measures (reduction of 0.04 and 0.02 p.p. per year, on average, respectively).

**Figure 2:**
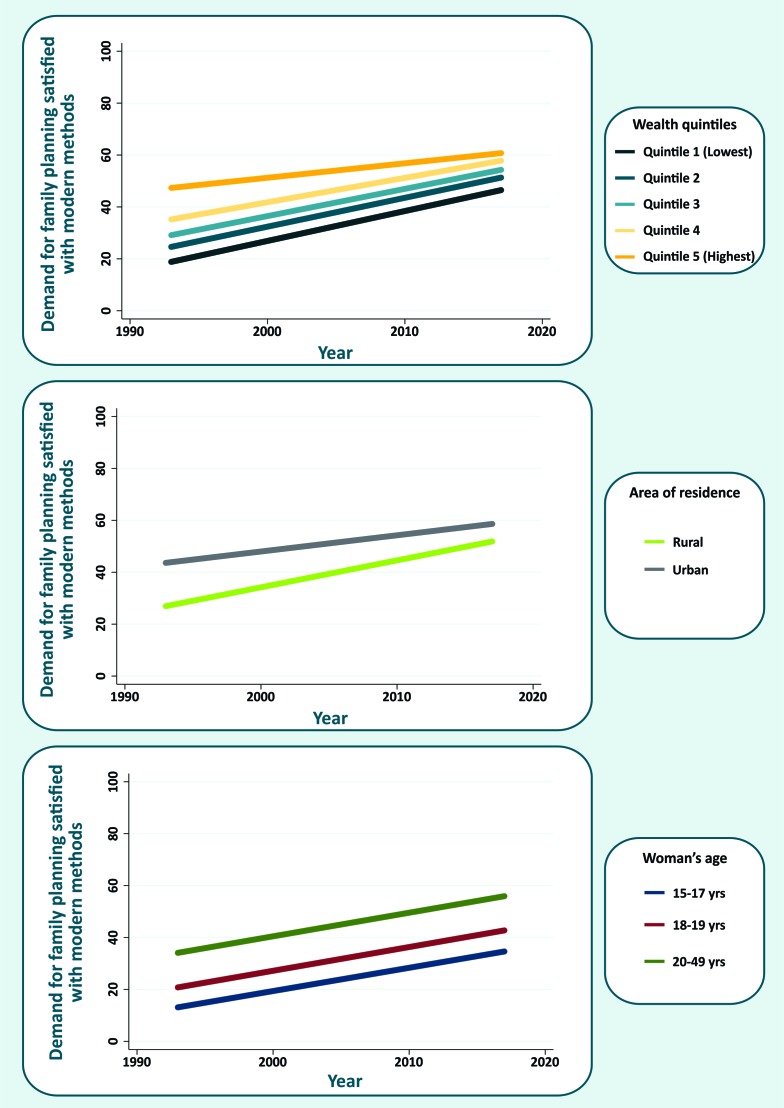
Global trends in demand for family planning satisfied with modern methods (mDFPS) according to women’s age, wealth quintiles and area of residence.

The wealthiest quintile (Q5) presented the slowest progress while the others had approximately the same rate of increase in mDFPS (**Figure 2**). Despite the important reduction in absolute inequalities according to area of residence, substantial differences remain. Women living in urban areas still present higher mDFPS than those living in rural settings. According to women’s age, we did not see the same pattern of closing gaps observed for wealth and area of residence. Polling all countries together, we found a similar increase in mDFPS for all age groups.

**Figure 3:**
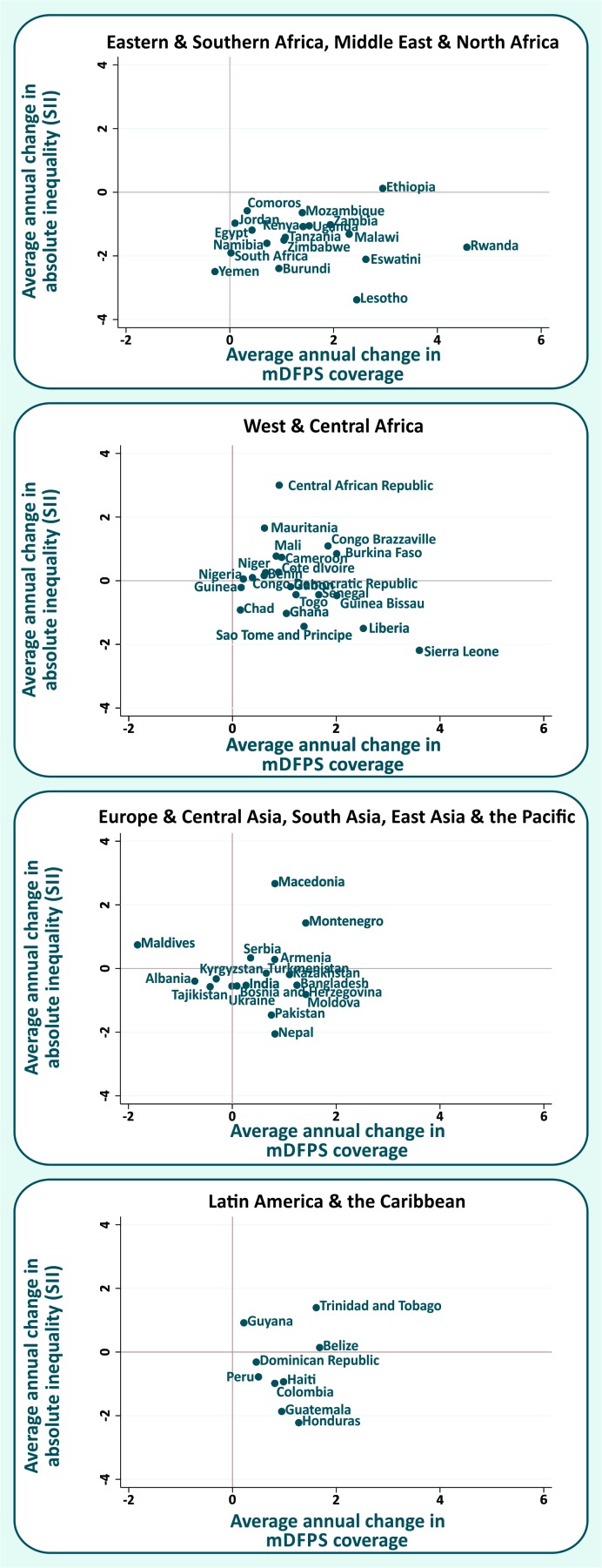
Average annual change in demand for family planning satisfied with modern methods (mDFPS) vs average annual change in the slope index of inequality (SII) in mDFPS. Some regions were merged given the small number of countries.

A comparison between the AAACs in mDFPS and in the absolute inequalities (SII) for each country, according to world, is presented in **Figure 3**. Most countries are in the bottom-right quadrant, which represents the best scenario, with increasing mDFPS coverage and diminishing absolute inequalities in the period. Notable countries in this situation are Sierra Leone (West & Central Africa), Lesotho, Rwanda and Eswatini (Eastern & Southern Africa), with the highest reductions in absolute inequality and highest increases in mDFPS. In the upper-right quadrant we have the countries where mDFPS coverage increased over time, but in a faster way for the wealthiest quintiles, increasing the inequalities. Among these countries, the highest increase in SII was observed in Central African Republic (West & Central Africa). Countries in the bottom-left quadrant have reduced both mDFPS coverage and absolute inequality. Countries in this situation are Tajikistan, Albania and Kyrgyzstan (Europe & Central Asia)(where only the change in mDFPS was significant),, Indonesia (East Asia & Pacific) (where only the change in SII was significant), and Yemen (Middle East & North Africa) (which had a significant change only in SII).

**Figure 4:**
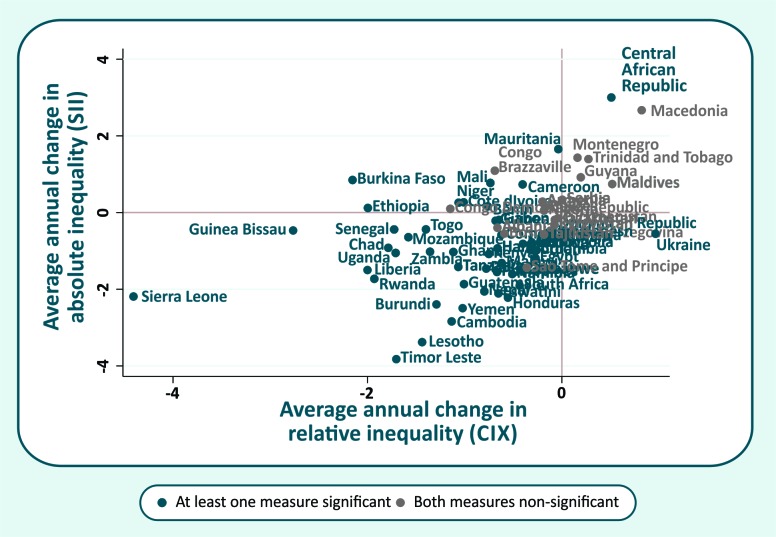
Average annual change in the concentration index of inequality (CIX) in demand for family planning satisfied with modern methods (mDFPS) vs average annual change in the slope index of inequality (SII) in mDFPS.

A comparison of the AAAC in both absolute and relative inequalities is shown in **Figure 4**. Countries in which both measures changed significantly (*P* < 0.05) are presented in blue. In this case, the best scenario is given by the bottom-left quadrant, which comprises countries that managed to reduce both relative and absolute inequalities. While most of countries are placed in this quadrant, many of them presented changes that are not significant in statistical terms for both measures (countries presented in grey). We highlighted six countries with the best performance in reducing inequalities: Sierra Leone, Guinea Bissau, Timor Leste, Rwanda, Lesotho, and Liberia. In the upper-right quadrant are the countries where both relative and absolute inequalities have increased in the period, representing the worse situation in terms of inequality. This is the case of Central African Republic (significant change only in the SII).

As an additional analysis, we estimated the AAAC in mDFPS and in wealth-based inequalities considering all sexually active women. For all practical purposes, the results presented the same picture. There are a few countries where the difference between the indicators is important: Guinea Bissau, Cameroon, Gabon, Montenegro, Serbia and Sierra Leone. Countries that presented high differences (above 10 p.p.) are Guinea Bissau and Serbia (Table S3 in **Online Supplementary Document**).

## DISCUSSION

Based on data from a large number of surveys carried out using similar methodology and analyzed in a standardized way, we presented an overview of trends in mDFPS in LMICs, including trends in inequalities of mDFPS according to key stratifiers. Overall, we present a positive picture, with increases in coverage and poor-rich gaps narrowing between 1993 and 2017. The countries from Eastern & Southern Africa stand out as those with the fastest increase in mDFPS in the period, while the slowest progress was observed for Middle East & North Africa. In relation to wealth-based inequalities in coverage, our results indicated that all world regions managed to reduce absolute inequalities, except Europe & Central Asia, which has low fertility levels [25,39] and a relatively low level of inequality in DFPS since the 1990s, and West & Central Africa, which presented the greatest wealth inequality in DFPS [26].

Eastern & Southern Africa and West & Central Africa presented a fast increase in mDFPS, what can partly be explained by the fact that it is easier to increase coverage where is it low at baseline. West & Central Africa has the lowest proportion of women in need of contraception compared to the other regions [40], and among those in need, less than half of those using contraception chose a modern method. Fear of opposition and stigmatization, laws which may require husband’s permission to access contraception, lack of knowledge about family planning, and limited method mix are some factors that limit the use of contraception [40]. Social norms are still important barriers in Eastern & Southern Africa. However, efforts have been made to increase the availability of different contraceptive methods and to promote knowledge of family planning, specially through school- and community-based programs with the intent of reducing resistance to contraceptive use [41].

At global level, we analyzed progress in mDFPS according to wealth quintiles, area of residence, and woman’s age. Our results indicated faster increase in rural areas, indicating that the gap is quickly closing and global differences between urban-rural areas could soon be very small. Unsatisfied demand for family planning can be partly explained by lack of access to sexual and reproductive services and products, which is usually more of a problem in rural settings. However, in countries going through fast urbanization, urban-rural inequalities may be underestimated given recently moved women might keep their more restrictive views towards contraception for some time.

Absolute differences between the quintiles are also reducing overtime given the slower progress pattern presented by the wealthiest quintile. No evident difference between the four lowest quintiles was observed in the global analysis. At regional level, faster increase in DFPS among women from disadvantage groups (from lower socioeconomic groups, rural areas, and with no or low levels of education) was already documented in the literature, mainly in Eastern & Southern Africa. The only exception is West & Central Africa where inequalities by wealth and area of residence increased in the least years [30].

At country level, mDFPS has increased much faster among individuals from lower socioeconomic positions in most of the countries analyzed, reducing both absolute and relative wealth-based inequalities. Some countries are increasing coverage among all population subgroups but inequalities remain, while inequalities increased in others, such as Central African Republic, from West & Central Africa and with mDFPS less than 30%. There is no single explanation to explain observed trends in all countries. Inequalities are expected to be small if the national coverage is very low or very high. On the other hand, it tends to increase as coverage increases, since the first groups to benefit are the wealthier and urban population. Finally, when coverage reaches high levels, inequality will decrease given most of the population is now covered [42].

Middle East & North Africa, Europe & Central Asia, and East Asia & Pacific were the regions with slowest progress in the period. We have information only about six countries in East Asia & Pacific, among which Indonesia, Mongolia, and Vietnam already presented high level of coverage in the 1990s or 2000s. Cambodia still has less than 60% coverage of mDFPS, however, coverage increased at a satisfactory pace and wealth-based inequalities decreased. Countries with low coverage and slow progress were Timor Leste and the Philippines (mDFPS of 46% and 56%, respectively, and average absolute annual change less than 1 percentage point). In Middle East & North Africa, three of the four countries included on our analyses presented mDFPS less than 60%, with almost no change in coverage over time. Egypt, the exception in the region, has low levels of women’s empowerment and high rates of population growth [43]. It presents mDFPS of 80% and relatively low levels of socioeconomic inequalities in mDFPS, but demand for family planning is still strongly determined by more conservative religious and social norms that put pressure on early marriage and childbearing [43].

West & Central Africa had the lowest level of mDFPS during the 1990s, and despite presenting the second fastest rate of progress, it still is the region with the lowest level of coverage in the 2010s, with an average mDFPS of 33% [25]. Only 3 of the 21 countries studied are likely to achieve 75% of mDFPS by 2030 (Burkina Faso, Liberia, and Sierra Leone). Sierra Leone was the only country in the region to present a more expressive increase in mDFPS, above 3 percentage points a year, as a result of the importance given by the government to equitable access to family planning services in its post-conflict agenda [44]. This country also achieved an important decrease of wealth-based inequality in mDFPS. The countries in West & Central Africa that need special attention are Chad and Congo Democratic Republic, given they faced decades of pronatalist policies and keep very low levels of mDFPS (14%), with almost no change over time.

The fastest increase in mDFPS among all countries studied was observed in Rwanda (Eastern & Southern Africa). From a mDFPS of 9% in 2000, the country achieved 64% of coverage in 2014, largely due to the *2006-2010 National FP Policy and Five-Year Strategy*, launched by the government to achieve the United Nations Millennium Development Goals [45]. The policy involved promoting family planning at all levels, encouraging family planning beyond cultural or religious objections, and providing a full range of contraceptive methods on public and private health services and on community-based family planning services [45]. Along with the increase in coverage, Rwanda presented a decrease of absolute wealth-based inequality in mDFPS, similar levels of modern contraceptive use in urban and rural areas, and a decrease of total fertility rate [46]. On the other extreme within the same region, Comoros presented a slow pace in mDFPS change, with low current levels of coverage (26%).

At regional level, model-based projections indicated that in 2030 demand for family planning satisfied by any contraceptive method will be around 80% or higher in most regions, except in Sub-Saharan Africa, Middle Africa, Western Africa, Melanesia, Micronesia, and Polynesia [47]. Despite the high fertility rate and the social norms against contraception in Africa regions [48], the government of many countries are committing with international organizations and initiatives to increase contraceptive use [49]. Projections can be outdated by such recent efforts. One example is Mozambique, where the government has committed with the Family Planning 2020 initiative in 2012 and since it, many efforts were launched to increase access to family planning, especially among adolescents and rural living families [50]. mDFPS was 30% in 2011 and the country achieve a much faster progress since it, reaching almost 50% of coverage in 2015.

Several indicators are used to evaluate population use of contraceptive methods. Extensively used indicators in the family planning field are contraception prevalence rate and unmet need for contraception. However, the standard definitions of these indicators have all women in reproductive age in its denominator, not taking into account the women’s intentions in terms of childbearing. Demand for family planning satisfied is an indicator that takes in its denominator only women that are fecund, sexually active and express the desire to delay or avoid pregnancy, being strictly a coverage indicator in the sense of measuring uptake of an intervention by those in need. In this sense, it reflects more precisely the performance of family planning policies and programs [5]. The definition of demand for family planning satisfy was revised in 2012, aiming to handle inconsistencies, improve the robustness of the indicator and reduce the number of questions needed in surveys. The changes proposed did not change the essence of the indicator, neither implicated in important numeric differences between the previous and the revised definitions [34]. Wherever data was available, we used the revised definition. Otherwise we used the previous one which does not differ significantly from the new one in numeric terms [34]. Despite being theoretically superior to contraceptive use, for instance, DFPS is based on several questions regarding fecundity and desire for more children, and thus can be affected by measurement error (especially in defining infecund women) and social desirability bias.

We adopted a definition of modern contraceptive methods that does not include lactational amenorrhea and fertility awareness methods, differently from a definition recently endorsed by WHO [51,52]. The main reason is that, particularly in LMICs, a large proportion of women present low levels of education and empowerment, and limited access to information. Women in such scenarios are likely to experience important difficulties in understanding and applying methods that depend on biological mechanisms perceptions and on periodic abstinence of sexual intercourse [53]. The focus on modern methods is important given their high individual effectiveness, although traditional methods can clearly help decrease population fertility rates, as we observed in countries such as Albania. mDFPS is a key measure to track progress in sexual and reproductive health, and it is one of the strengths of this work [5].

A limitation of our analysis is the restriction of our indicator to women married or in a union. Ideally, all sexually active women should be taken into account [4,18]. But 24 countries would be left out of the main analyses if we chose this approach. We present the analyses for all sexually active women in the supplementary material for those countries with information. No important differences were observed comparing the results for partnered women only and all sexually active women. Typically, larger differences arise in the youngest group of women, where most unmarried sexually active women are.

It should be noted that despite the large number of countries included in our analyses, it includes only those with at least two surveys from DHS or MICS, carried out since 1993. Results at regional level must be interpreted carefully, especially for Middle East & North Africa, East Asia & Pacific, and for Latin America & Caribbean where we were able to include less than half of the countries in each region. Our results were not weighted by population size, and thus represent the average situation of the countries in each region, each country having the same weight, independent of population size, which prevents large countries to dominate the results. We restrict the analysis to countries with data available since 2010 to increase comparability, however, countries have differences in the period covered that need to be interpreted with caution.

## CONCLUSION

Efforts to increase mDFPS coverage and decrease inequalities have succeeded in most of the countries included in our analysis. However, some of them still present lower coverage levels than desired and slow progress over time. Our results pointed to a set of countries that are unlikely to reach high coverage of mDPFS by 2030 and where more efforts must be directed to family planning programs taking into account context and particularities of each country and population subgroup in order to allow women to plan their pregnancies according their wishes.

## Additional material

Online Supplementary Document

Pelotas, RS Brazil 96020-220 fhellwig@equidade.org
