# Supplementary material for: Time trends in demand for family planning satisfied: analysis of 73 countries using national health surveys over a 24-year period
Source: J Glob Health. 2019 Oct 22;9(2):020423. doi: 10.7189/jogh.09.020423 (PMC6820067; doi:10.7189/jogh.09.020423)
Supplement: Online Supplementary Document [file jogh-09-020423-s001.pdf]

Supplementary Material

Table S1. List of countries with available data on mDFPS trends between 1993 and 2016 for married women.

Region	ISO	Country	Year
Middle East & North Africa	EGY	Egypt	1995, 2000, 2005, 2008, 2014
	IRQ	Iraq	2006*, 2011*
	JOR	Jordan	1997, 2002, 2007, 2012, 2017
	YEM	Yemen	2006*, 2013
West & Central Africa	BEN	Benin	1996, 2001, 2006, 2011, 2014*
	BFA	Burkina Faso	1998, 2003, 2006*, 2010
	CAF	CAR	1994, 2006*, 2010*
	CMR	Cameroon	1998, 2004, 2011, 2014*
	TCD	Chad	1996, 2004, 2010*, 2014
	COG	Congo Brazzaville	2005, 2011, 2014*
	COD	Congo Democratic Republic	2007, 2010*, 2013
	CIV	Cote d'Ivoire	1994, 1998, 2006, 2011, 2016*
	GAB	Gabon	2000, 2012
	GHA	Ghana	1993, 1998, 2003, 2008, 2011*, 2014
	GIN	Guinea	1999, 2005, 2012, 2016*
	GNB	Guinea Bissau	2006*, 2014*
	LBR	Liberia	2007, 2013
	MLI	Mali	1995, 2001, 2006, 2009*, 2012, 2015*
	MRT	Mauritania	2007*, 2011*, 2015*
	NER	Niger	1998, 2006, 2012
	NGA	Nigeria	1999, 2003, 2007*, 2008, 2011*, 2013, 2016*
	STP	Sao Tome and Principe	2008, 2014*
	SEN	Senegal	1997, 2005, 2010, 2012, 2014, 2015, 2016, 2017
	SLE	Sierra Leone	2008, 2010*, 2013
	TGO	Togo	1998, 2006*, 2010*, 2013
Eastern & Southern Africa	BDI	Burundi	2010, 2016
	COM	Comoros	1996, 2012
	SWZ	Eswatini	2006, 2010*, 2014*
	ETH	Ethiopia	2000, 2005, 2011, 2016
	KEN	Kenya	1993, 1998, 2003, 2008, 2014
	LSO	Lesotho	2004, 2009, 2014
	MWI	Malawi	2000, 2004, 2010, 2013, 2015
	MOZ	Mozambique	1997, 2003, 2011, 2015
	NAM	Namibia	2000, 2006, 2013
	RWA	Rwanda	2000, 2005, 2010, 2014
	ZAF	South Africa	1998, 2016
	TZA	Tanzania	1996, 1999, 2004, 2010, 2015
	UGA	Uganda	1995, 2000, 2006, 2011, 2016
	ZMB	Zambia	1996, 2001, 2007, 2013

	ZWE	Zimbabwe	1994, 1999, 2005, 2010, 2014*, 2015
Europe & Central Asia	ALB	Albania	2008, 2017
	ARM	Armenia	2000, 2005, 2010, 2015
	BIH	Bosnia and Herzegovina	2006*, 2011*
	KAZ	Kazakhstan	1995, 1999, 2010*, 2015*
	KGZ	Kyrgyzstan	1997, 2005*, 2012, 2014*
	MKD	Macedonia	2005*, 2011*
	MDA	Moldova	2005, 2012*
	MNE	Montenegro	2005*, 2013*
	SRB	Serbia	2005*, 2010*, 2014*
	TJK	Tajikistan	2005*, 2012, 2017
	TKM	Turkmenistan	2006*, 2015*
	UKR	Ukraine	2005*, 2007, 2012*
South Asia	BGD	Bangladesh	1993, 1996, 1999, 2004, 2007, 2011, 2012*, 2014
	IND	India	1998, 2005, 2015
	MDV	Madives	2009, 2016
	NPL	Nepal	1996, 2001, 2006, 2010*, 2011, 2014*, 2016
	PAK	Pakistan	2006, 2012, 2017
East Asia & Pacific	KHM	Cambodia	2000, 2005, 2010, 2014
	IDN	Indonesia	1994, 1997, 2002, 2007, 2012
	MNG	Mongolia	2005*, 2010*, 2013*
	PHL	Philippines	1993, 1998, 2003, 2008, 2013, 2017
	TLS	Timor Leste	2009, 2016
	VNM	Vietnam	1997, 2002, 2010*, 2013*
Latin America & the Caribbean	BLZ	Belize	2006*, 2011*, 2015*
	COL	Colombia	1995, 2000, 2005, 2010, 2015
	CUB	Cuba	2006*, 2010*, 2014*
	DOM	Dominican Republic	1996, 1999, 2002, 2007, 2013, 2014*
	GTM	Guatemala	1995, 1998, 2014
	GUY	Guyana	2006*, 2009, 2014*
	HTI	Haiti	1994, 2000, 2005, 2012, 2016
	HND	Honduras	2005, 2011
	PER	Peru	1996, 2000, 2004, 2005, 2006, 2007, 2008, 2009, 2010, 2011, 2012, 2014, 2015, 2016
	TTO	Trinidad and Tobago	2006*, 2011*

* following the year represents a MICS survey, all the others are DHS surveys.

Table S2. Average annual absolute change (AAAC) in demand for family planning satisfied with modern methods (mDFPS), in absolute (slope index of inequality, SII) and in relative inequality (concentration index, CIX) by world region.

Region	Unweighted AAAC			Weighted AAAC		
	**mDFPS (p value)**	**SII (p value)**	**CIX (p value)**	**mDFPS (p value)**	**SII (p value)**	**CIX (p value)**
Middle East & North Africa	0.21 (0.131)	-0.69 (<0.001)	-0.47 (<0.001)	-0.37 (0.509)	-0.75 (0.302)	-0.12 (0.724)
West & Central Africa	0.96 (<0.001)	-0.04 (<0.001)	-0.10 (<0.001)	1.02 (<0.001)	0.12 (0.623)	-0.96 (<0.001)
Eastern & Southern Africa	1.50 (<0.001)	-1.17 (<0.001)	-1.10 (<0.001)	1.51 (<0.001)	-1.34 (<0.001)	-1.13 (<0.001)
Europe & Central Asia	0.46 (0.016)	-0.02 (0.900)	-0.05 (0.565)	-0.28 (0.681)	0.11 (0.784)	0.04 (0.869)
South Asia	0.68 (0.001)	-0.91 (<0.001)6	-0.32 (<0.001)	0.03 (0.934)	-0.58 (0.107)	-0.19 (0.139)
East Asia & Pacific	0.60 (<0.001)	-1.4 (<0.001)	-0.55 (<0.001)	0.11 (0.827)	-1.34 (<0.001)	-0.50 (<0.001)
Latin America & Caribbean	0.74 (<0.001)	-0.87 (<0.001)	-0.46 (<0.001)	0.68 (0.049)	-0.87 (0.002)	-0.47 (<0.001)
All countries	0.91 (<0.001)	-0.69 (<0.001)	-0.47 (<0.001)	0.64 (0.002)	-0.63 (<0.001)	-0.65 (<0.001)

Table S3. Demand for family planning satisfied with modern methods among both, only partnered and all sexually active women (ordered according to differences in coverage).

Region	Country	Year	Source	mDFPS (%)	
				Married women	Sexually active women
West & Central Africa	Guinea Bissau	2006	MICS	18.3	29.2
		2014	MICS	34.4	50.1
Europe & Central Asia	Serbia	2005	MICS	29.6	35.1
		2010	MICS	35.5	49.1
West & Central Africa	Cameroon	1998	DHS	18.2	20.7
		2004	DHS	27.0	30.7
		2011	DHS	30.1	34.9
		2014	MICS	36.4	46.3
West & Central Africa	Sierra Leone	2008	DHS	17.0	19.7
		2010	MICS	26.6	33.4
		2013	DHS	35.3	43.5
Europe & Central Asia	Montenegro	2005	MICS	29.2	30.7
		2013	MICS	40.5	47.6
West & Central Africa	Gabon	2000	DHS	19.4	24.4
		2012	DHS	32.8	39.6
Europe & Central Asia	Bosnia and Herzegovina	2006	MICS	22.5	24.6
		2011	MICS	23.0	29.0
Eastern & Southern Africa	Mozambique	1997	DHS	18.1	19.7
		2003	DHS	26.7	31.9
		2011	DHS	31.4	33.5
		2015	DHS	48.0	50.3
West & Central Africa	Guinea	1999	DHS	13.9	16.1
		2005	DHS	13.0	16.4
		2012	DHS	11.3	16.3
		2016	MICS	22.1	24.6
West & Central Africa	Cote d'Ivoire	1994	DHS	11.2	15.3
		1998	DHS	17.0	21.5
		2011	DHS	25.6	28.2
		2016	MICS	31.3	33.0
Eastern & Southern Africa	Namibia	2006	DHS	70.7	74.9
		2000	DHS	64.7	65.8
		2013	DHS	74.7	78.1
LAC	Paraguay	2016	MICS	86.4	82.5
West & Central Africa	Nigeria	1999	DHS	26.1	28.4
		2003	DHS	23.3	26.8
		2007	MICS	30.1	32.6
		2008	DHS	23.3	26.9
		2011	MICS	29.9	32.6
		2013	DHS	28.6	32.7
		2016	MICS	26.4	26.8
West & Central Africa	Congo Brazzaville	2005	DHS	19.9	22.6
		2011	DHS	29.1	32.9
		2014	MICS	37.1	39.2

Europe & Central Asia	Ukraine	2007	DHS	61.6	65.4
		2012	MICS	69.4	70.7
West & Central Africa	Togo	1998	DHS	12.5	14.7
		2006	MICS	19.8	23.0
		2010	MICS	25.5	27.9
		2013	DHS	32.2	34.1
West & Central Africa	Congo Democratic Republic	2007	DHS	12.9	15.8
		2010	MICS	12.5	13.4
		2013	DHS	14.8	16.2
LAC	Costa Rica	2011	MICS	89.3	86.8
Eastern & Southern Africa	Angola	2015	DHS	23.8	26.8
West & Central Africa	Burkina Faso	1998	DHS	12.8	15.5
		2003	DHS	20.3	22.8
		2006	MICS	27.9	29.1
		2010	DHS	36.6	38.1
Eastern & Southern Africa	Uganda	1995	DHS	17.8	19.0
		2000	DHS	24.4	26.6
		2006	DHS	29.0	31.5
		2011	DHS	40.2	41.3
		2016	DHS	49.7	50.4
LAC	Dominican Republic	1996	DHS	76.2	75.2
		1999	DHS	77.2	76.9
		2002	DHS	79.7	78.5
		2007	DHS	82.6	80.9
		2013	DHS	82.4	80.5
		2014	MICS	85.2	82.9
West & Central Africa	Liberia	2007	DHS	21.8	24.0
		2013	DHS	37.0	38.3
LAC	Guyana	2009	DHS	56.0	57.9
		2014	MICS	52.4	51.6
East Asia & Pacific	Mongolia	2010	MICS	79.9	78.4
		2013	MICS	74.9	73.0
West & Central Africa	Central African Republic	1994	DHS	10.4	12.3
		2006	MICS	24.0	24.2
		2010	MICS	23.8	24.6
West & Central Africa	Benin	1996	DHS	8.1	9.0
		2001	DHS	14.8	15.5
		2006	DHS	12.8	14.6
		2011	DHS	16.3	18.0
Eastern & Southern Africa	South Africa	1998	DHS	77.4	77.6
		2016	DHS	77.8	75.7
LAC	Belize	2011	MICS	73.1	71.4
		2015	MICS	67.1	64.8
LAC	Mexico	2015	MICS	85.8	82.9
LAC	Panama	2013	MICS	76.4	74.5
Eastern & Southern Africa	Zambia	1996	DHS	28.8	29.0
		2001	DHS	36.8	37.2
		2007	DHS	39.4	40.2
		2013	DHS	62.4	60.7

Eastern & Southern Africa	Comoros	1996	DHS	20.5	21.9
		2012	DHS	25.8	26.2
Eastern & Southern Africa	Tanzania	1996	DHS	31.9	33.0
		1999	DHS	35.7	36.5
		2004	DHS	39.3	40.6
		2010	DHS	46.9	48.3
		2015	DHS	52.1	52.9
West & Central Africa	Mali	1995	DHS	14.8	16.2
		2001	DHS	15.7	16.3
		2006	DHS	16.0	16.2
		2009	MICS	22.4	22.7
		2012	DHS	27.1	27.7
		2015	MICS	34.0	34.7
West & Central Africa	Ghana	1993	DHS	17.8	17.8
		1998	DHS	23.1	23.6
		2003	DHS	31.0	31.7
		2008	DHS	28.2	29.4
		2011	MICS	40.0	41.4
		2014	DHS	38.4	37.7
LAC	Peru	1996	DHS	50.8	50.8
		2000	DHS	60.3	59.6
		2004	DHS	55.8	55.8
		2005	DHS	57.5	58.6
		2006	DHS	57.7	58.0
		2007	DHS	56.1	56.5
		2008	DHS	57.9	59.3
		2009	DHS	59.5	60.3
		2010	DHS	59.7	60.6
		2011	DHS	60.2	61.4
		2012	DHS	61.0	62.0
		2014	DHS	61.9	62.6
		2015	DHS	64.0	64.9
		2016	DHS	64.5	65.3
West & Central Africa	Chad	1996	DHS	8.4	9.4
		2004	DHS	5.4	5.8
		2010	MICS	5.6	6.9
		2014	DHS	14.0	14.7
Eastern & Southern Africa	Malawi	2000	DHS	45.5	45.1
		2004	DHS	45.6	45.0
		2010	DHS	58.5	58.3
		2013	MICS	74.8	74.0
		2015	DHS	74.4	73.2
Eastern & Southern Africa	Eswatini	2006	DHS	62.9	63.9
		2010	MICS	81.9	83.0
		2014	MICS	85.3	85.8
West & Central Africa	Sao Tome and Principe	2008	DHS	43.8	44.9
		2014	MICS	52.1	51.5
Eastern & Southern Africa	Ethiopia	2000	DHS	14.6	15.7
		2005	DHS	28.2	28.4
		2011	DHS	49.7	50.2

		2016	DHS	59.5	59.6
Eastern & Southern Africa	Rwanda	2000	DHS	8.8	9.2
		2005	DHS	16.3	16.1
		2010	DHS	60.8	60.4
		2014	DHS	64.3	63.3
LAC	Colombia	1995	DHS	71.5	70.8
		2000	DHS	73.4	72.7
		2005	DHS	78.4	77.3
		2010	DHS	83.5	83.0
		2015	DHS	86.5	85.6
Europe & Central Asia	Kosovo	2013	MICS	21.0	22.3
Europe & Central Asia	Belarus	2012	MICS	76.0	77.0
West & Central Africa	Niger	1998	DHS	18.5	19.5
		2006	DHS	18.5	18.7
		2012	DHS	27.7	27.8
Europe & Central Asia	Kazakhstan	1995	DHS	61.5	61.2
		1999	DHS	70.2	69.6
		2010	MICS	81.4	81.4
		2015	MICS	85.4	84.6
Eastern & Southern Africa	Madagascar	1997	DHS	21.7	21.1
		2003	DHS	33.0	32.9
		2008	DHS	47.9	47.0
LAC	Haiti	1994	DHS	21.3	21.8
		2000	DHS	32.9	33.2
		2005	DHS	34.0	34.1
		2012	DHS	44.2	43.5
		2016	DHS	43.1	41.8
West & Central Africa	Gambia	2013	DHS	23.8	24.5
LAC	Bolivia	1994	DHS	24.0	24.1
		1998	DHS	33.9	34.3
		2003	DHS	31.9	32.4
		2008	DHS	41.7	42.4
Eastern & Southern Africa	Lesotho	2004	DHS	51.4	52.1
		2009	DHS	65.2	65.3
		2014	DHS	76.1	76.3
Europe & Central Asia	Albania	2008	DHS	12.5	13.1
		2017	DHS	6.0	6.1
LAC	El Salvador	2014	MICS	85.0	83.2
Eastern & Southern Africa	Zimbabwe	1994	DHS	64.7	64.7
		1999	DHS	72.6	72.2
		2005	DHS	78.4	78.0
		2010	DHS	78.1	77.8
		2014	MICS	88.1	87.5
		2015	DHS	84.9	84.4
Europe & Central Asia	Kyrgyzstan	1997	DHS	68.7	68.6
		2005	MICS	74.3	74.4
		2012	DHS	61.5	61.0
East Asia & Pacific	Lao	2011	MICS	68.6	68.0
Eastern & Southern	Kenya	1993	DHS	40.0	40.0
		1998	DHS	48.5	48.1

Africa		2003	DHS	47.9	48.1
		2008	DHS	54.8	54.7
		2014	DHS	70.5	70.2
LAC	Brazil	1996	DHS	81.9	81.5
Europe & Central Asia	Moldova	2005	DHS	56.6	56.2
		2012	MICS	66.5	66.3
West & Central Africa	Senegal	1997	DHS	17.0	17.0
		2005	DHS	23.1	23.4
		2010	DHS	27.5	27.6
		2012	DHS	33.8	33.9
		2014	DHS	42.2	42.6
		2015	DHS	43.2	43.4
		2016	DHS	45.2	45.3
East Asia & Pacific	Philippines	1993	DHS	36.4	36.4
		1998	DHS	40.3	40.3
		2003	DHS	48.0	47.8
		2008	DHS	45.9	45.6
		2013	DHS	50.8	50.4
		2017	DHS	56.0	55.3
East Asia & Pacific	Timor Leste	2009	DHS	38.8	38.8
		2016	DHS	45.8	45.4
LAC	Cuba	2010	MICS	89.7	89.8
		2014	MICS	89.7	89.5
LAC	Barbados	2012	MICS	70.7	70.9
LAC	Trinidad and Tobago	2006	MICS	56.2	56.3
		2011	MICS	64.3	61.0
Europe & Central Asia	Armenia	2000	DHS	27.7	27.6
		2005	DHS	28.6	28.7
		2010	DHS	38.1	38.2
		2015	DHS	38.9	39.1
LAC	Honduras	2005	DHS	68.4	68.5
		2011	DHS	76.0	75.9
Europe & Central Asia	Uzbekistan	1996	DHS	74.1	74.0
		2006	MICS	83.0	83.0
Eastern & Southern Africa	Burundi	2010	DHS	32.6	32.7
		2016	DHS	38.3	38.5
South Asia	Bhutan	2010	MICS	85.8	85.6
LAC	Guatemala	1995	DHS	47.5	47.4
		1998	DHS	49.6	49.7
		2014	DHS	65.3	65.4
East Asia & Pacific	Cambodia	2000	DHS	34.6	34.6
		2005	DHS	41.6	41.6
		2010	DHS	51.4	51.4
		2014	DHS	56.1	56.0
East Asia & Pacific	Vietnam	2010	MICS	75.1	75.0
East Asia & Pacific	Myanmar	2015	DHS	74.8	74.7
LAC	St. Lucia	2012	MICS	72.5	72.3
East Asia & Pacific	Indonesia	2012	DHS	78.9	78.9
South Asia	India	2005	DHS	69.9	69.9
		2015	DHS	71.8	71.8

Europe & Central Asia	Azerbaijan	2006	DHS	17.8	17.8
Europe & Central Asia	Macedonia	2005	MICS	21.7	21.7
South Asia	Nepal	2006	DHS	60.9	60.9
		2011	DHS	55.9	55.9
		2016	DHS	56.0	56.0
Europe & Central Asia	Tajikistan	2012	DHS	50.6	50.6
		2017	DHS	50.4	50.4

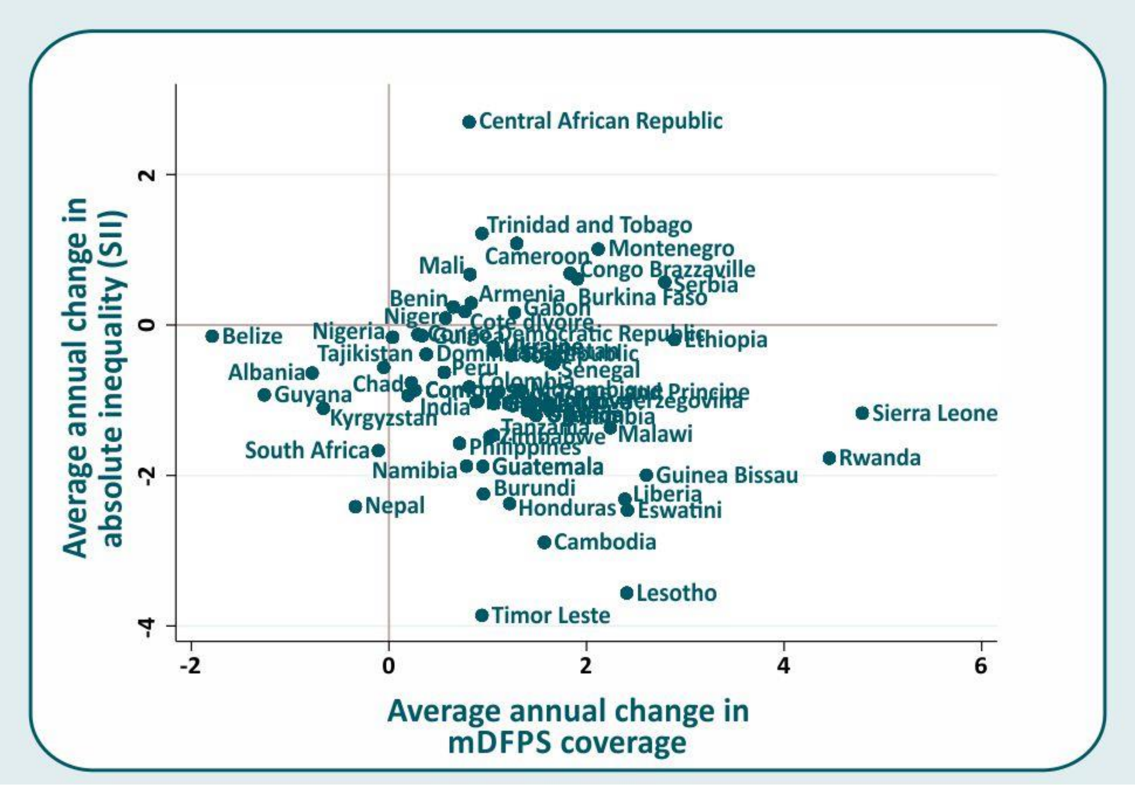

Figure S1. Average annual change in demand for family planning satisfied with modern methods (mDFPS) vs. average annual change in the slope index of inequality (SII) in mDFPS (all sexually active women).

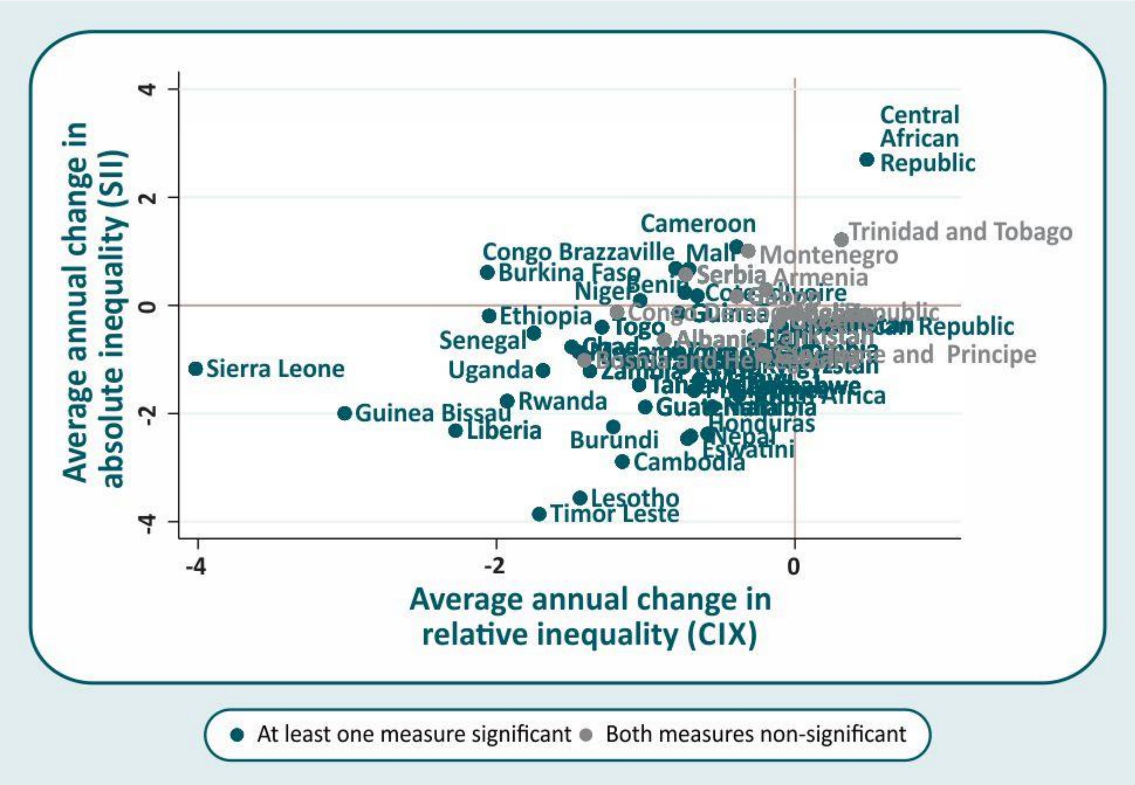

Figure S2. Trends in demand for family planning satisfied with modern methods (mDFPS) and in absolute inequality (SII) in mDFPS according to world region. Average annual change in the concentration index of inequality in demand for family planning satisfied with modern methods (mDFPS) vs. average annual change in the slope index of inequality (SII) in mDFPS (all sexually active women).
